# Differential Regulation of Cellular FAM111B by Human Adenovirus C Type 5 E1 Oncogenes

**DOI:** 10.3390/v13061015

**Published:** 2021-05-28

**Authors:** Wing-Hang Ip, Britta Wilkens, Anastasia Solomatina, Judith Martin, Michael Melling, Paloma Hidalgo, Luca D. Bertzbach, Thomas Speiseder, Thomas Dobner

**Affiliations:** Department of Viral Transformation, Leibniz Institute for Experimental Virology (HPI), 20251 Hamburg, Germany; britta.wilkens@leibniz-hpi.de (B.W.); a.solomatina@uni-bonn.de (A.S.); judith_martin@gmx.net (J.M.); mickamell@outlook.de (M.M.); paloma.hidalgo@leibniz-hpi.de (P.H.); luca.bertzbach@leibniz-hpi.de (L.D.B.); thomas.speiseder@gmx.net (T.S.)

**Keywords:** human adenovirus, FAM111 trypsin-like peptidase B, adenovirus early region 1 (E1), E1A, E1B-55K, E4orf6, viral replication, antiviral host factor

## Abstract

The adenovirus type 5 (HAdV-C5) E1 transcription unit encodes regulatory proteins that are essential for viral replication and transformation. Among these, E1A and E1B-55K act as key multifunctional HAdV-C5 proteins involved in various steps of the viral replication cycle and in virus-induced cell transformation. In this context, HAdV-C5-mediated dysregulations of cellular factors such as the tumor suppressors p53 and pRB have been intensively investigated. However, cellular components of downstream events that could affect infection and viral transformation are widely unknown. We recently observed that cellular FAM111B is highly regulated in an E1A-dependent fashion. Intriguingly, previous reports suggest that FAM111B might play roles in tumorigenesis, but its exact functions are not known to date. Here, we set out to investigate the role of FAM111B in HAdV-C5 infections. We found that (i) FAM111B levels are upregulated early and downregulated late during infection, that (ii) FAM111B expression is differentially regulated, that (iii) FAM111B expression levels depend on the presence of E1B-55K and E4orf6 and that (iv) a FAM111B knockdown increases HAdV-C5 replication. Our data indicate that FAM111B acts as an anti-adenoviral host factor that is involved in host cell defense mechanisms in productive HAdV-C5 infection. Moreover, these findings suggest that FAM111B might play an important role in the host antiviral immune response that is counteracted by HAdV-C5 E1B-55K and E4orf6 oncoproteins.

## 1. Introduction

Human adenoviruses (HAdVs) are non-enveloped, linear dsDNA viruses that mostly cause asymptomatic or mild disease in younger and immunocompetent individuals. However, HAdV infections of immunocompromised patients or patients with pre-existing respiratory or cardiac conditions can cause severe disease courses and are serious health issues [1]. Therefore, it is important to thoroughly study basic mechanisms of HAdV infection as well as the interplay between HAdV gene products and host immunity.

In a yet unpublished transcriptome analysis, we found that cellular FAM111B was strongly upregulated upon transduction with the two HAdV-C5 oncogenes E1A and E1B. The exact function of FAM111B, the second and last member of the “family with sequence similarity 111” gene family, is widely unknown. Interestingly though, a recent study demonstrated that FAM111B co-precipitates with HAdV-C5 E1B-55K [2]. FAM111B contains a predicted trypsin-like cysteine/serine peptidase domain, comprising of two subdomains connected by a linker region. Notably, three specific alterations within the linker region were previously found to induce hereditary fibrosing poikiloderma [3]. Mercier et al. showed that FAM111B is important for fibrosis development, which is a key pathological process in a variety of human diseases [3]. The trypsin-like cysteine/serine peptidase domain of FAM111B shares 45% homology with FAM111A. Mutations in this region were identified to induce Kenny–Caffey syndrome and osteocraniostenosis, which are characterized by impaired skeletal development [4,5]. Intriguingly, the 11q12 locus (and the genes encoding FAM111A and FAM111B in this region, specifically) is associated with prostate cancer susceptibility and serves as a signature to discriminate the risk of metastases [6]. In line with this, more and more recent evidence continues to point towards an involvement of FAM111B in tumorigenesis [7,8,9]. Finally, the SV40 virus large T antigen interacts with FAM111A to inhibit viral replication in restrictive cells and FAM111A restricts vaccinia virus (VACV) replication [10,11,12,13]. However, it was also found that SV40 virus still replicates in a restrictive cell line upon FAM111A depletion [10].

In this report, we set out to investigate the role of FAM111B in the context of HAdV-C5 infection and the regulation of this putative cellular oncogene by HAdV-C5 E1 genes.

## 2. Materials and Methods

### 2.1. Cells and Culture Conditions

H1299 (ATCC no. CRL-5803: ATCC Global Bioresource Center, Manassas, Virginia, VA, USA) and A549 (ACC 107; DSMZ-German Collection of Microorganisms and Cell Cultures, Braunschweig, Germany) were grown in DMEM (Gibco; Carlsbad, CA, USA) supplemented with 10% FCS, 100 U/mL penicillin and 100 µg/mL streptomycin. All cells were kept in incubators at 37 °C in 5% CO_2_. FAM111B knockdown cell lines were generated by lentiviral transduction of A549 cells. Transduced cells were maintained under 2 µM puromycin selection. All cells were tested for mycoplasma contamination on a regular basis.

### 2.2. Plasmids and Transient Transfections

HAdV-C5 E1B, HA-E1A (wild type (wt), RB-, CBP- and RB/CBP-binding deficient) and E2F-1 were expressed from pcDNA plasmids (Invitrogen, Carlsbad, CA, USA) under CMV IE1-control. For transient transfections, subconfluent H1299 cells were incubated with DNA and 25-kDa linear polyethylenimine (PEI) as described previously [14]. For the short hairpin RNA (shRNA)-mediated FAM111B knockdown, we purchased shRNAs against FAM111B targeting the coding strand sequence 5′-GCCTGCCTAGTGATTCTCATT-3′ (mission shRNA, clone ID NM_198947.1-638s1c1; Sigma-Aldrich, St. Louis, MO, USA) based on the viral pLKO.1-puro vector. The shFAM111B plasmids were co-transfected with lentiviral packaging plasmids by calcium phosphate transfection according to the manufacturer’s protocol (ProFection Mammalian Transfection System; Promega, Madison, WI, USA).

### 2.3. Luciferase Reporter Assays

For dual luciferase assays, subconfluent H1299 cells were transfected with 0.2 µg reporter (pLightSwitch-FAM111B prom; Product ID: S707175; SwitchGear Genomics, Carlsbad, CA, USA), 0.1 µg pFirefly-TK (expresses firefly luciferase under the control of the HSV-TK promoter) and 0.6 µg of effector plasmids (HAdV-C5 HA-E1A (wt, RB-, CBP-, RB/CBP-binding deficient), E1B-55K and E2F-1 as well as LeGO-iBLB2 E1B-55K and LeGO-iVLN2 E1A [15]) by PEI transfection as described above. Cell extracts were prepared 24 h post transfection (h p.t.) and renilla activity was recorded with a luminometer (Lumat LB9510, Berthold Technologies GmbH & Co.KG, Bad Wildbad, Germany).

### 2.4. Viruses

H5*pg*4100 harboring deletions in the E3-coding region served as the wt HAdV-C5 virus [16]. The mutant virus H5*pm*4149 (4x) harbors four stop codons within the E1B coding region and H5*pm*4154 (E4orf6-) has a stop codon at P66 of E4orf6 to abrogate translation of the respective proteins [17,18]. Viruses were propagated, titrated and used for infections as described previously [19].

Virus yield was analyzed at indicated time points by quantitative E2A immunofluorescence staining. Viral titers were determined as described before and are represented as the number of fluorescence-forming units (FFU)/µL [20].

### 2.5. Antibodies and Protein Analysis

Primary monoclonal and polyclonal antibodies used to detect viral proteins included mouse monoclonal E1A (M73), E1B-55K (2A6), E4orf6 (RSA3), E2A (B6-8), rat monoclonal L4-100K (6B10) and HAdV-C5 rabbit polyclonal serum L133 [21]. Primary monoclonal and polyclonal antibodies specific for cellular and ectopically expressed proteins included rabbit polyclonal FAM111B (HPA038637; Sigma-Aldrich) and mouse monoclonal *β*-actin (AC-15; Sigma-Aldrich). Horseradish peroxidase (HRP)-conjugated secondary antibodies anti-rabbit IgG, anti-mouse IgG and anti-rat IgG for detection of proteins by immunoblotting were obtained from Jackson/Dianova (Hamburg, Germany).

All protein extracts were incubated in radioimmunoprecipitation assay (RIPA) lysis buffer on ice for 30 min. Total cell lysates were sonicated in a high-intensity cup horn (Branson Ultrasonics, Brookfield, CT, USA) for 45 s (40 pulses; output, 0.6 and 0.8 impulses/second) before insoluble debris was removed by centrifugation (11,000 rpm, 3 min, 4 °C). Protein concentration was measured photometrically with Bradford reagent (Bio-Rad, Hercules, CA, USA). Equal amounts of total protein were separated on 10% SDS-polyacrylamide gels after denaturation (5× SDS sample buffer, 95 °C, 3 min) and subjected to immunoblotting exactly as previously described [21].

### 2.6. In Vitro Translation and Pulldown Assays

FLAG-tagged FAM111B was in vitro translated with the TNT Coupled Wheat Germ Systems (VWR, Radnor, PA, USA) according to the manufacturer’s instruction. In vitro-translated FLAG-tagged FAM111B was purified by immunoprecipitation (IP) with an ANTI-FLAG M2 Affinity Gel (Sigma-Aldrich). Briefly, FLAG-tagged FAM111B was added to the supplied beads together with TBS buffer and protease inhibitors (1% phenylmethylsulfonyl fluoride (PMSF), 0.1% aprotinin, 1 µg/mL leupeptin, 1 µg/mL pepstatin, 25 mM iodacetamide and 25 mM N-ethylmaleimide) and incubated overnight at 4 °C. An aliquot of FLAG-tagged FAM111B was eluted from the beads using 2× Laemmli buffer as controls. The remaining beads coupled with FLAG-tagged FAM111B were incubated with protein lysates prepared in RIPA buffer as described in the previous section for 2 h at 4 °C. Bound proteins were eluted with 2× Laemmli buffer at 95 °C for 5 min.

### 2.7. Indirect Immunofluorescence

For indirect immunofluorescence analyses, 1.5 × 10^5^ A549 cells were grown on glass coverslips in 6-well cell dishes one day prior to infection. Cells were fixed with paraformaldehyde (PFA; 4% (*v*/*v*) in PBS) at room temperature (RT) for 20 min. PFA-fixed cells were permeabilized with phosphate-buffered saline (PBS) supplemented with 0.5% Triton X-100 for 10 min at room temperature and blocked with 1.5 mL Tris-buffered saline-BG (TBS-BG; BG represents 5% (*w*/*v*) BSA and 5% (*w*/*v*) glycine) for 1 h at RT. Next, coverslips were incubated with the indicated primary antibody diluted in PBS in a humidity chamber for 30 min. The corresponding secondary antibody (Alexa 488- (Invitrogen), Cy3 (Jackson, West Grove, PA, USA)-conjugated secondary antibodies) diluted in PBS was added afterwards. Finally, nuclei were stained with DAPI (4,6-diaminidino-2-phenylindole) in PBS (1:1000, (*v*/*v*) from 1 mg/mL stock) for 5 min, before the cover slips were mounted in mounting solution (Energene, Regensburg, Germany). Images were acquired using a confocal spinning-disk microscope (Nikon Eclipse Ti-E stand (Nikon, Tokyo, Japan); Yokogawa CSU-W1 spinning disk (Yokogawa, Tokyo, Japan; 2× Andor888 EM-CCD camera (Oxford Instruments, Abingdon, UK); Nikon 100× NA 1.49 objective (Nikon)).

### 2.8. Isolation and Quantification of Nucleic Acids

RNAs were isolated by incubating fresh cell pellets (4 × 10^6^ cells) in 600 µL TRIzol (ThermoFisher, Waltham, MA, USA) and 60 µL of 1-bromo-3-chloropropane for 10 min at RT. Samples were then shaken and centrifuged at 12,000× *g* for 15 min at 4 °C. The aqueous phase with nucleic acids was precipitated with 500 µL of isopropanol (12,000× *g*, 10 min, 4 °C), washed with 1 mL ethanol (75%, *v*/*v*) by vortexing and pelleted (7500× *g*, 5 min, 4 °C). The resulting pellets were air-dried for 5–10 min and resuspended in 80 µL RDD buffer containing 10 µL RNase-free DNase I (Qiagen, Hilden, Germany) to digest traces of the remaining DNA for 30 min at RT. DNase I was heat-inactivated (75 °C, 5 min) followed by RNA precipitation with RNase-free LiCl solution (final concentration, 2.5 M; Applied Biosystems, Foster City, CA, USA) for 30 min at −20 °C. Samples were then centrifuged (16,000× *g* for 20 min at 4 °C) and RNAs were washed with ice-cold ethanol (75%, *v*/*v*). After air-drying for 5–10 min, the RNA was dissolved for 10 min in 5–50 µL nuclease-free water (Qiagen). RNA was transcribed into cDNA using a reverse transcription system (Applied Biosystems) according to the manufacturer’s protocol using random primers. cDNAs were quantified by real-time quantitative PCR (qPCR) using SYBR green reagents (SensiMix Plus SYBR; Quantace, London, UK), FAM111B and GAPDH-specific primers (FAM111B forward: 5′-GCCCTTGAAATGCAGAATCCA-3′; reverse: 5′-GCTGTAAACACACTACGGTCTAA-3′ and GAPDH forward: 5′-ACCACAGTCCATGCCATCAC-3′; reverse: 5′-TCCACCACCCTGTTGCTGTA-3′) and a Rotor-Gene cycler (Corbett Life Sciences, Sydney, Australia). FAM111B mRNA levels were calculated relative to the level of cellular GAPDH RNA.

## 3. Results and Discussion

### 3.1. FAM111B RNA and Protein Levels Are Upregulated in HAdV-C5-Infections

**Figure 1:**
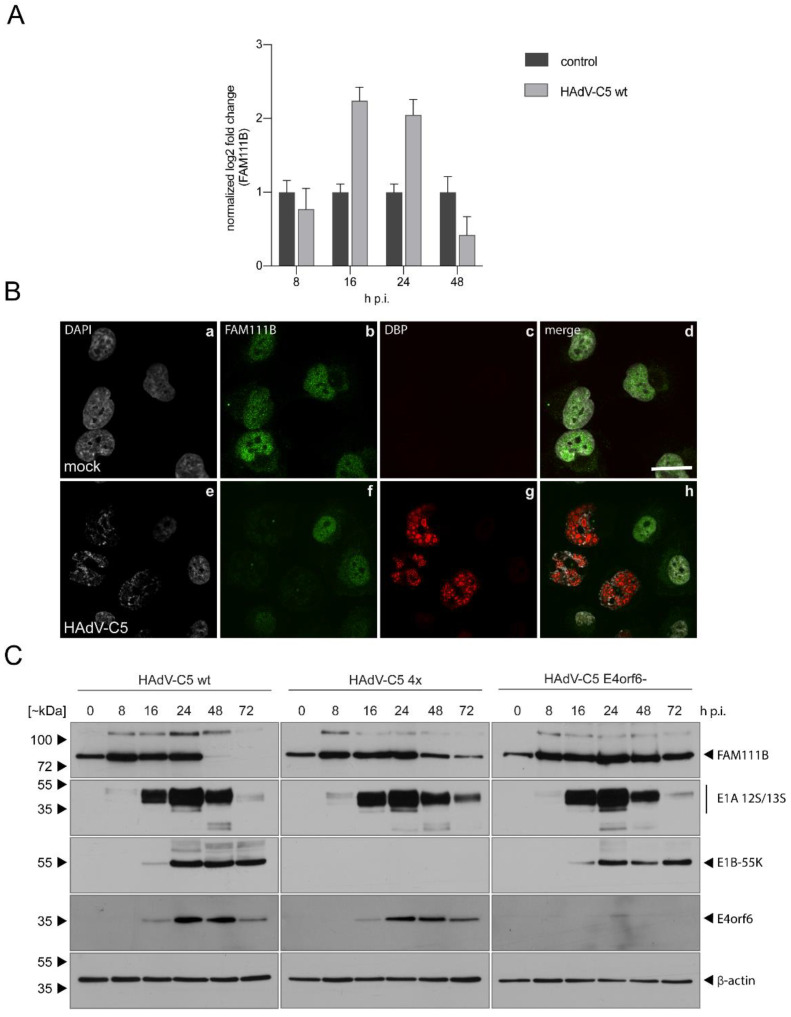
FAM111B transcript and protein levels are differentially regulated during infection. Subconfluent (**A**,**B**) A549 cells or (**C**) H1299 cells were left uninfected (mock) or infected with HAdV-C5 (MOI 20) and harvested at indicated time points. (**A**) Total RNA was extracted and reverse transcribed and resulting cDNA was amplified with primer pairs specific for FAM111B or cellular GAPDH by RT-PCR (ΔΔCt). The experiment was done in two triplicates. Representative FAM111B mRNA amounts are shown normalized to GAPDH. Error bars indicate standard error of mean. (**B**) Cells were fixed with 4% PFA at 24 h p.i. DBP was visualized as infection control with a mouse mAb B6-8 (α-DBP). FAM111B was visualized using the rabbit FAM111B pAb HPA038637. Primary antibodies were detected with secondary antibodies conjugated to Alexa 488 (green) or Alexa 555 (red), and nuclei were stained with DAPI. Representative staining patterns of >30 analyzed cells and overlays of the single images are shown. The scale bar in (d) corresponds to 10 µm. (**C**) H1299 cells were infected with wt HAdV-C5, E1B-55K-deleted (4×) and E4orf6-deleted (6-) viruses (MOI 20) and harvested at indicated time points. Total cell lysates were resolved by SDS-PAGE and visualized by immunoblotting. Proteins were detected using pAb HPA038637 (α-FAM111B), mAb M73 (α-E1A), mAb 2A6 (α-E1B-55K), mAb RSA3 (α-E4orf6) and mAb AC-15 (*β*-actin). Molecular masses in kilodaltons (kDa) are indicated on the left, while corresponding proteins are indicated on the right. Mock is indicated as 0 h p.i.

To analyze whether FAM111B transcript and protein levels are differentially regulated during HAdV-C5 infection, we infected the human lung adenocarcinoma cell line A549, that is widely used in HAdV research, with wt HAdV-C5 [16] and first investigated mRNA levels of FAM111B during infection at different time points post infection (Figure 1A). FAM111B transcript levels increased at 16–24 h post infection (h p.i.) compared to mock (Figure 1A) but interestingly dropped at 48 h p.i. (Figure 1A). These results indicate that FAM111B is regulated at the transcriptional level in response to HAdV-C5 infection, implying that FAM111B might belong to the family of immediate early response genes activated upon HAdV infection.

To check whether this is also reflected in FAM111B protein levels, we analyzed FAM111B protein expression by immunofluorescence. We stained HAdV- and mock-infected A549 cells with specific antibodies detecting FAM111B and the viral protein DBP (E2A), which formed nuclear spherical structures representing the sites of viral replication [22]. FAM111B showed diffuse nuclear staining in mock-infected cells excluding the nucleoli and a weak cytoplasmic signal, while FAM111B intensity is reduced in HAdV-C5-infected cells (Figure 1B). However, these findings are in contrast to the elevated FAM111B transcript levels at 24 h p.i. (Figure 1A). We therefore performed a time-resolved analysis to investigate FAM111B protein expression levels during a course of 8–72 h p.i. by immunoblotting (Figure 1C). Here, FAM111B levels increased early, but decreased later during infection—observations that are consistent with our previous mRNA analyses. The steady increase in FAM111B protein levels at 8–24 h was followed by a decrease at 24–72 h (Figure 1C).

These results indicate that FAM111B expression is upregulated early in infection. In contrast, FAM111B protein levels are downregulated during the late course of infection. Complete abrogation of FAM111B protein expression late during wt HAdV-C5 infection could point towards potent post-translational regulations that remain to be elucidated. Taken together, these data show that wt HAdV-C5 infection strongly induced mRNA and protein levels of FAM111B at early time points, which decreased in the late phase of the infection.

### 3.2. FAM111B Expression Levels Are Differentially Regulated by E1A, E1B-55K and E4orf6

Adenovirus E4orf6 and E1B-55K proteins form a functional cullin-based E3 ubiquitin ligase complex to target a number of cellular proteins for proteasomal degradation [18,23,24]. Within the complex, E4orf6 associates via multiple BC boxes with cellular elongins B and C to enable binding of either Cul5 or Cul2 and further components to build up the core ligase complex, and E1B-55K serves as a substrate recognition factor. This complex is formed to degrade cellular proteins, which would otherwise counteract viral replication [25,26,27]. To investigate whether E1B and E4orf6 mediate the degradation of FAM111B during late times of infection, we used HAdV-C5 mutants that do not express E1B-55K or E4orf6 [17,18] (Figure 1C). As infection control and control for the virus mutants, we stained for E1A, E1B-55K and E4orf6 and could detect viral proteins starting at 16 h p.i. While FAM111B protein levels of wt-infected cells were not detectable at 48–72 h p.i., they were substantially reduced in cells infected with virus mutants lacking E1B-55K or E4orf6, most likely due to decreased transcript levels at these stages of infection (Figure 1C). These data suggest that the E3 ubiquitin ligase complex formed by E1B and E4orf6 together with cellular proteins targets FAM111B at late time points of infection.

**Figure 2:**
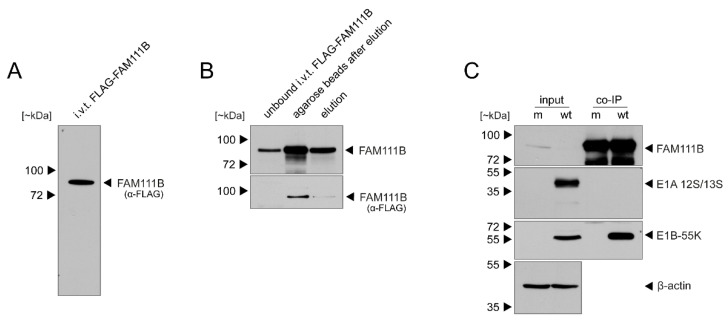
In vitro-translated FAM111B interacts with E1B-55K. (**A**) FLAG-tagged FAM111B was produced by in vitro transcription/translation (i.v.t.) with wheat germ extract. (**B**) Samples were coupled with anti-FLAG M2 affinity gels. (**C**) Subconfluent A549 cells were infected with wt HAdV-C5 (MOI 5) and total cell lysates were prepared at 48 h p.i. and incubated with FLAG-tagged FAM111B beads. Input cell lysates and FAM111B co-immunoprecipitated proteins were resolved by SDS-PAGE and visualized by immunoblotting. Proteins were detected using pAb HPA038637 (α-FAM111B), mAb M73 (α-E1A), mAb 2A6 (α-E1B-55K) and mAb AC-15 (*β*-actin). Molecular masses in kilodaltons (kDa) are indicated on the left and corresponding proteins are indicated on the right.

Since E1B-55K serves as a substrate recognition factor to target cellular antiviral proteins for proteasomal degradation, we then tested whether E1B-55K interacts with FAM111B. We first in vitro translated FLAG-FAM111B (Figure 2A) and immobilized FAM111B using FLAG antibody-bound agarose beads. Although a considerable amount of FAM111B was still bound on the agarose beads, the eluate was positive for FAM111B after staining with a FAM111B-specific monoclonal antibody (mAb) (Figure 2B). For IPs, HAdV-C5-infected or mock-infected A549 whole-cell lysates were incubated with immobilized FLAG-tagged FAM111B. Immunoblot analysis of E1A 12S/13S and E1B-55K proteins after co-IP with the FAM111B-specific mAb revealed that FAM111B interacts only with E1B-55K but not with E1A 12S/13S (Figure 2C), supporting the idea that FAM111B interacts with E1B-55K and serves as a novel substrate of the viral E3 ubiquitin ligase complex. These data are supported by work from Hung et al., who have previously identified FAM111B as a binding partner of the viral E1B-55K protein [2].

**Figure 3:**
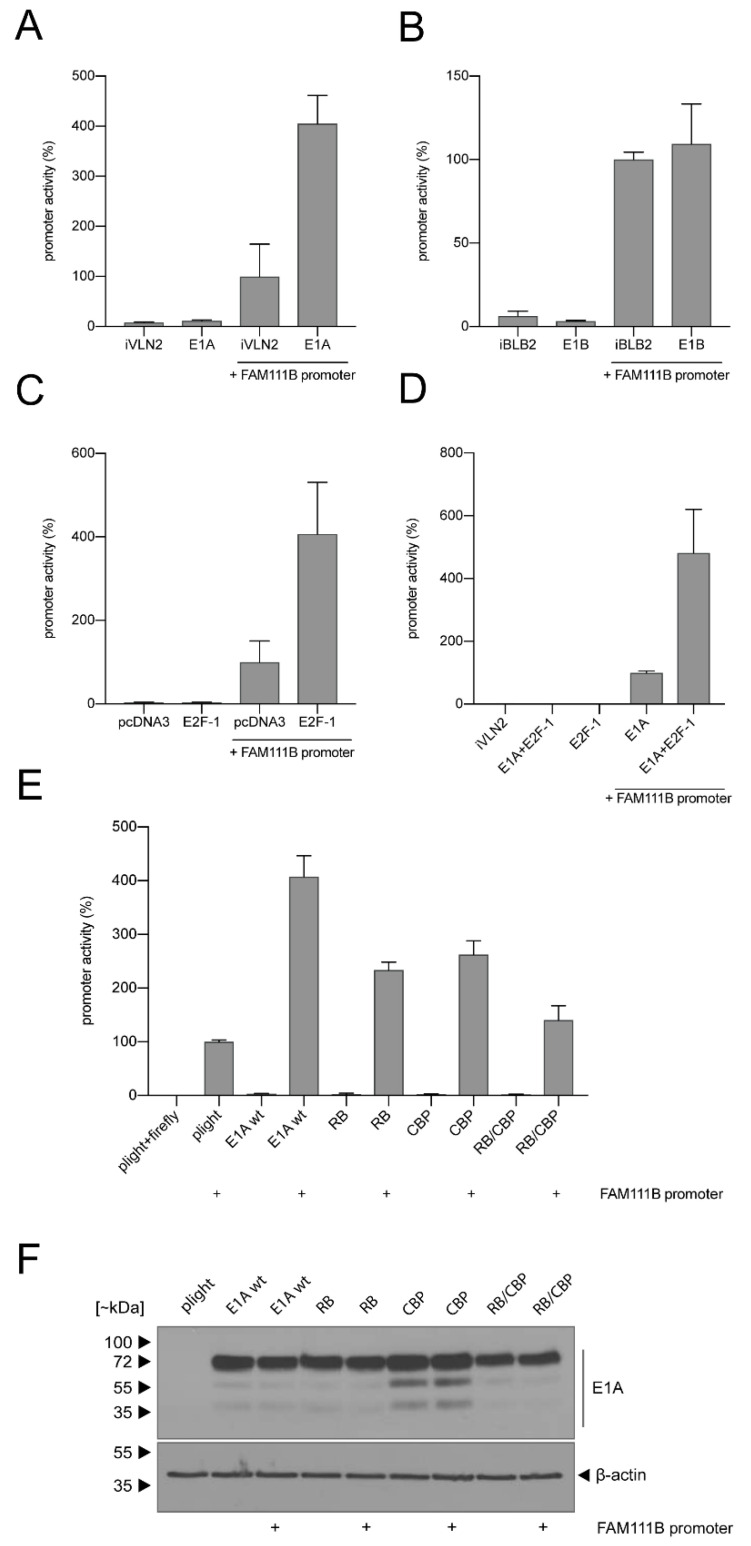
E1A activates FAM111B transcription that is enhanced by E2F-1 and depends on its RB/CBP motif. H1299 cells were transfected with 0.5 µg of pFirefly-TK, 0.5 µg of FAM111B promoter. (**A**) LeGO-iVLN2 E1A, (**B**) LeGO-iBLB2 E1B-55K, (**C**) E2F-1, (**D**) LeGO-iVLN2 E1A and E2F-1, (E) LeGO-iVLN2 wt E1A, RB, CBP single- or RB/CBP double-binding mutants. (F) Input levels of total cell lysates of (**E**) were detected using mouse mAb M-58 (α-E1A) and mAb AC-15 (*β*-actin). Luciferase activities were determined at 24 h p.t. with total cell extracts. Firefly luciferase activities are shown in percentages. (**A**–**E**) Representative means and standard deviations are shown. Experiments were done in two triplicates.

To analyze transcriptional regulation of FAM111B by E1A and E1B, we performed luciferase reporter gene assays under control of the FAM111B promoter. Activity was measured after co-transfection with the respective constructs, demonstrating that FAM111B promoter activity is enhanced upon co-transfection with the E1A- but not the E1B-containing plasmid (Figure 3A,B). E1A is known to interact with pRB and its family members to activate E2F-dependent transcription and cell cycle entry, and transcriptional regulation occurs through CBP/p300-dependent genes by E1A [28,29]. Moreover, the FAM111B promoter is responsive to E2F-1, E2F-4 and CREB [30]. To investigate whether E1A activates the FAM111B promoter via the pRB/E2F pathway, we first analyzed responsiveness of the FAM111B promoter to E2F-1 (Figure 3C). Additionally, E1A was co-transfected to investigate whether it has an additive effect on FAM111B promoter activation (Figure 3D). We found that E2F-1 stimulated transcription of the FAM111B promoter approximately 4-fold higher compared to the FAM111B promoter alone (Figure 3C). Intriguingly, co-transfection of E2F-1 plus E1A further increased FAM111B promoter activity (Figure 3D).

Finally, we performed luciferase assays with E1A mutants deficient in binding to RB or CBP or both (Figure 3E). As expected, the FAM111B promoter was activated about 4-fold after co-transfection with wt E1A (Figure 3E), while functional inactivation of E1A-binding sites to RB, CBP or both clearly diminished E1A’s transcriptional stimulation of the FAM111B promoter (Figure 3E). To control for the expression levels of the different E1A mutants, whole cell lysates were immuno-stained with an E1A-specific antibody (Figure 3F). In summary, these results show that binding of E1A to p300/CBP and pRB is important for transcriptional regulation of FAM111B. While E1A seems to modulate FAM111B transcription, E1B might regulate protein levels.

### 3.3. FAM111B Knockdown Increases HAdV-C5 Gene Expression

**Figure 4:**
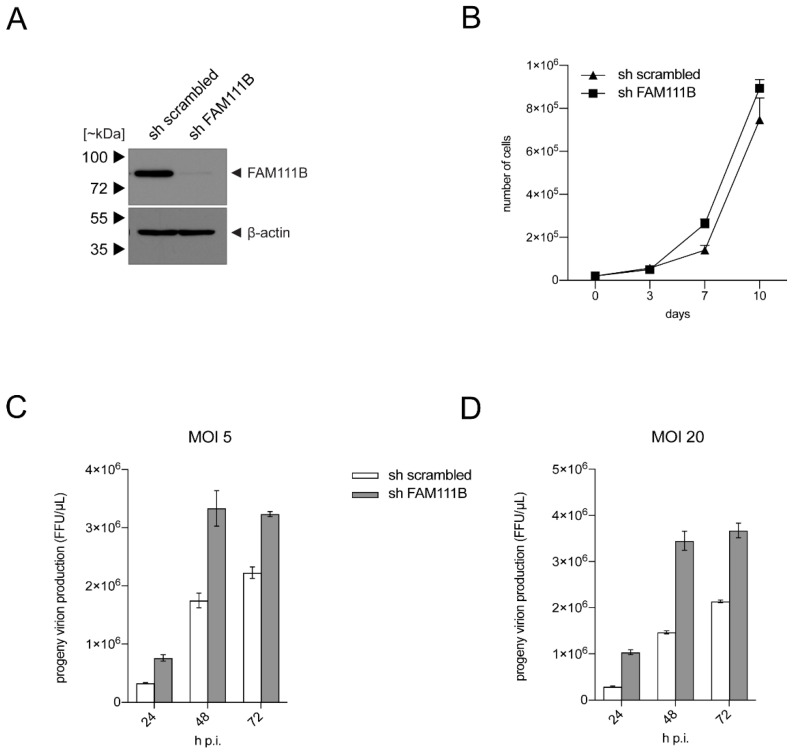
FAM111B depletion enhances viral progeny production. A549 cells were transduced with lentiviral particles harboring an shRNA against FAM111B and scrambled shRNA as control. Transduced cells were selected with puromycin from 48 h p.t. for 7 days. (**A**) An aliquot of transduced and positively selected cells were subjected to total cell extract preparation. Proteins were separated by SDS-PAGE and visualized by immunoblotting by applying pAb HPA038637 (α-FAM111B) and mAb AC-15 (*β*-actin). Molecular masses in kilodaltons (kDa) are indicated on the left and corresponding proteins on the right. (**B**) A total of 1 × 10^4^ cells were cultivated and cell growth was monitored by determining the absolute cell numbers at the indicated time points. Values show the mean of triplicate measurements of a representative experiment. The experiment was performed twice. Error bars represent the corresponding standard error of mean. (**C**) Cells were infected with HAdV-C5 at (**C**) MOI 5 and (**D**) MOI 20 and viral particles were harvested at the indicated time points after infection. Virus yield was determined by quantitative B6-8 (α-DBP) immunofluorescence staining on HEK 293 cells after methanol fixation. The experiments were performed twice in triplicate. A representative experiment is shown for each MOI. Error bars indicate the standard error of the mean.

Finally, we set out to analyze the role of FAM111B during HAdV-C5 infection in more detail. Therefore, we used shRNA knockdowns (KD) of FAM111B in A549 cells, in comparison to control cells that express scrambled shRNA (Figure 4A). First, the KD was confirmed by immunoblotting as FAM111B shRNA reduced steady-state levels of the FAM111B protein, while no effect in FAM111B levels was observed with the control shRNA (Figure 4A). Next, cell growth analyses revealed that A549s replicate slightly better with depleted FAM111B, although this was not statistically significant (Figure 4B). Finally, we infected A549-derived FAM111B KD cells and the respective control cell line with wt HAdV-C5 (MOI 5 and 20) and performed virus yield analysis at time points 24 h, 48 and 72 h p.i. (Figure 4C,D). The FAM111B KD enhanced progeny virus production by up to 3-fold at 48 h p.i. These results suggest that FAM111B may function as a host restriction factor that affects virus replication and virus progeny production.

## 4. Conclusions

This study demonstrates that FAM111B expression is transcriptionally activated at early times after HAdV-C5 infection. This upregulation is mediated by E1A on a transcriptional level, resulting in elevated FAM111B expression dependent on E1A’s CBP/RB-binding motifs in an E2F-dependent manner. While FAM111B protein levels increase early during infection, they are downregulated at later time points, likely mediated by the E3 ubiquitin ligase complex composed of E1B and E4orf6. Taken together, our data strongly indicate that FAM111B is a novel member of a growing list of cellular restriction factors that are activated in response to HAdV infection during the immediate early phase of the infection. This, together with the findings that the related FAM111A protein restricts SV40 and VACV replication, suggests that members of nuclear trypsin-like proteases act as restriction factors to antagonize replication of DNA viruses. Future investigations will aim to further reveal its impact on HAdV replication and also possible contributions of FAM111B to viral transformation. These data could be applied to other viral oncogenes and viral infections in general that manipulate key cellular pathways. Finally, deeper insights into the exact functions of antiviral restriction factors might potentially contribute to the identification of new therapeutic antiviral strategies.
